# Spatially fractionated radiation therapy (SFRT): a scoping review of dosimetric and biological evidence supporting a valley dose–driven translational framework

**DOI:** 10.1186/s13014-026-02865-2

**Published:** 2026-06-04

**Authors:** Suyan Bi, Changyou Zhong, Ying Long, Zhongqing Huang, Zhitao Dai

**Affiliations:** 1 Post-Doctoral Research Center, Yuebei People’s Hospital, Southern Medical University, Shaoguan, Guangdong Province China; 2 Radiotherapy Center, Meizhou People’s Hospital, Meizhou, Guangdong Province China; 3 Department of Medical Image Center, Yuebei People’s Hospital, Shantou University Medical College, Shantou, Guangdong Province China; 4 Department of Radiation Oncology, National Cancer Center, National Clinical Research Center for Cancer, Cancer Hospital & Shenzhen Hospital, Chinese Academy of Medical Sciences and Peking Union Medical College, Shenzhen, Guangdong Province 518116 China

**Keywords:** Spatially fractionated radiation therapy (SFRT), Valley dose, Peak-to-valley dose ratio (PVDR), Bulky tumors, Scoping review, GRID therapy, LATTICE radiotherapy

## Abstract

**Background:**

Spatially fractionated radiation therapy (SFRT)—mainly including GRID, LATTICE, minibeam, and microbeam—widens the therapeutic window for bulky or radioresistant tumors. Yet clinical translation faces two persistent hurdles: technical heterogeneity across modalities and a lack of evidence-based, tumor-specific dose prescription guidelines.

**Methods:**

This scoping review maps the SFRT evidence landscape from 2005 to 2025. Following PRISMA-ScR guidelines, we synthesized 60 studies (preclinical, clinical, technical) and identified valley dose (D_valley_) as a consistently reported strong candidate predictor of the therapeutic window.

**Results:**

Based on this pattern, we outline an evidence-informed organizational framework. Synthesizing the extracted data, we delineate a tripartite structure that emerged from the literature: (1) a tumor-and modality-specific dosimetric decision frame work (Level 1) capturing histology-dependent D_valley_ benchmarks (e.g., ≥ 2 Gy for cervical LATTICE, ~ 10 Gy for brain proton minibeam) with PVDR guidance; (2) a Mechanism- Biomarker-Outcome (MBO) chain (Level 2) linking spatial dose gradients to quantifiable cascades—vascular normalization (DCE-MRI Ktrans), immune preservation (CD8⁺ density), and bystander signaling (HMGB1, TNF-α, ROS); (3) an exploratory surrogate marker model (Level 3) identifying early short-term metrics (6-month metabolic response ≥ 50% SUVmax reduction, cytokine dynamics) as candidate correlates of long-term local control.

**Conclusion:**

By mapping the shift from peak-centric ablation to a valley-dose-driven approach reflected in the literature, this review provides a structured, evidence-informed scaffold for future clinical trial design and technical harmonization. The framework presented is intended as a hypothesis-generating tool to inform the standardized implementation of SFRT in precision radiation oncology.

**Supplementary Information:**

The online version contains supplementary material available at 10.1186/s13014-026-02865-2.

## Introduction

Spatially fractionated radiation therapy (SFRT) has re-emerged as a compelling strategy for malignancies that pose formidable challenges to conventional radiotherapy—namely, bulky lesions (≥ 4 cm in diameter), radioresistant histologies (e.g., glioblastoma exhibiting ≤ 50% 1-year local control post-standard treatment), and tumors situated in critical proximity to organs-at-risk (OARs) [1, 2]. Unlike uniform-dose irradiation, which frequently forces a compromise between tumor control probability (TCP) and normal tissue complication probability (NTCP), SFRT employs a highly heterogeneous dose distribution. By delivering alternating high-dose “peaks” and low-dose “valleys,” this spatial modulation is designed to sterilize macroscopic tumor foci while preserving normal tissue stem cell niches and, as emerging radiobiological evidence suggests, safeguarding immune effector cells within the low-dose corridors [3, 4].

Over the past two decades, various SFRT modalities have transitioned from theoretical physics to clinical and preclinical evaluation. These encompass a diverse range of modalities, such as megavoltage GRID therapy, modern LATTICE radiotherapy (LRT), microbeam radiotherapy (MRT), and proton- based minibeam radiotherapy (pMBRT) [5, 6]. The clinical signals have been highly encouraging: photon GRID has demonstrated an overall clinical response rate of approximately 78% in advanced malignancies, with particularly high palliative efficacy in soft tissue sarcomas [7], while proton minibeam configurations have reduced high-grade neurotoxicity to negligible levels in preclinical brain models [8].

While these outcomes are compelling, the widespread clinical translation of SFRT remains impeded by a fundamental dosimetric ambiguity: which parameter most consistently correlates with tumor response and normal tissue sparing? Historically, treatment planning has been profoundly peak-centric. The peak dose is typically prescribed to reflect direct tumor ablation, while the peak-to-valley dose ratio (PVDR) is optimized as a surrogate for dose heterogeneity [6–10]. However, this mathematical reliance on PVDR obscures the absolute value of the dose delivered to the interconnecting tissue—the valley dose (D_valley_).

Accumulating evidence across diverse cohorts suggests that this peak-centric paradigm may overlook a critical determinant of the therapeutic window. In recent preclinical evaluations, such as murine oral and glioma models, D_valley_ consistently showed stronger associations with efficacy than peak dose, often accounting for over 70% of the variance in tumor growth delay (R2 > 0.70) [11, 12]. A similar pattern is observed in the clinical domain; for instance, in bulky cervical tumors treated with LATTICE therapy, achieving a D_valley_ ≥2 Gy was associated with local control rates exceeding 80%—a metric notably superior to historical controls for such refractory cases [13]. Similarly, in pMBRT applications for central nervous system lesions, clinically relevant preclinical studies in glioma models have demonstrated that maintaining appropriate D_valley_ ceilings is critical for minimizing high-grade neurotoxicity and improving long-term prognosis compared with conventional proton therapy [14].

**Fig. 1:**
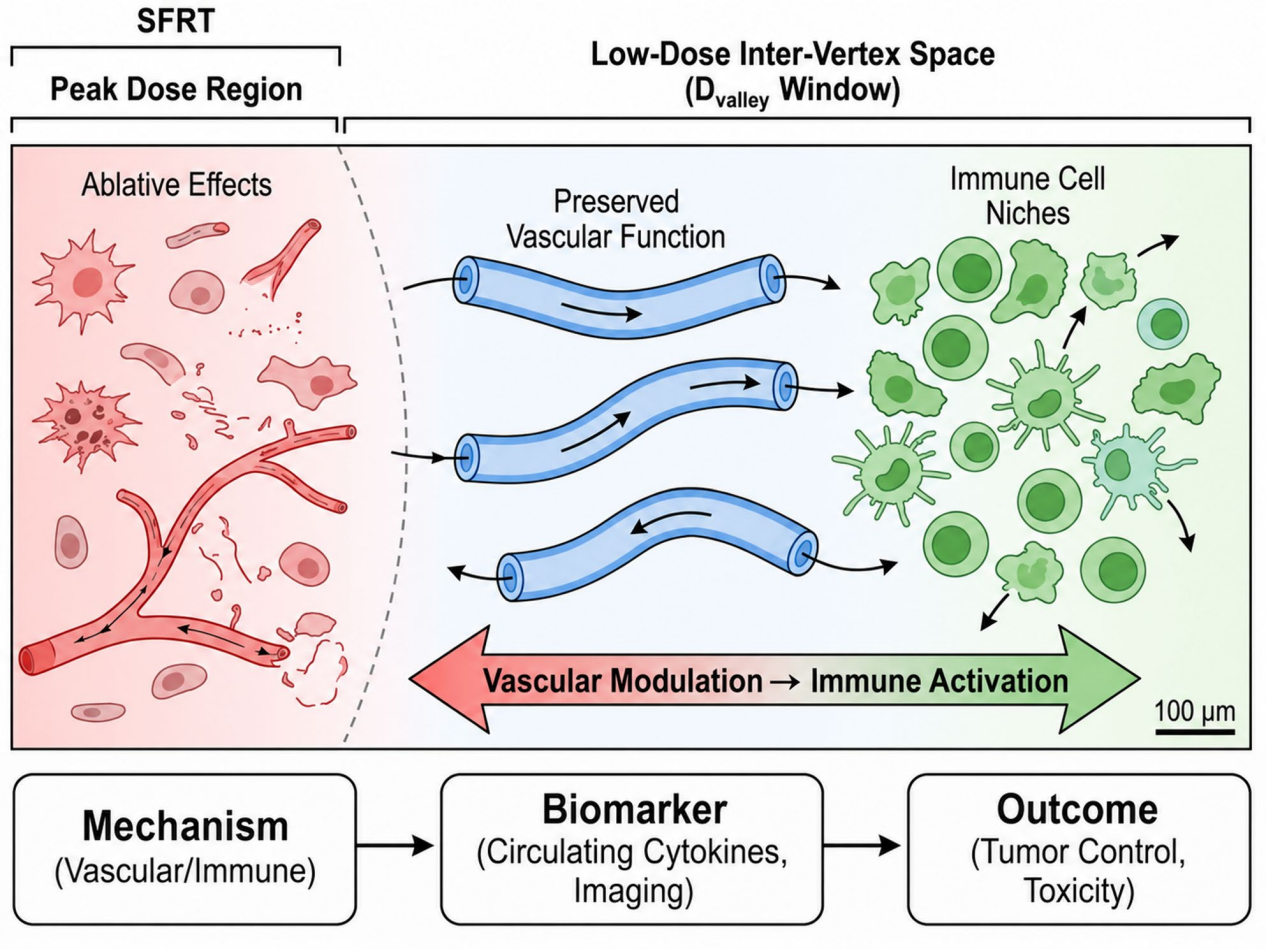
The Optimal D_valley_ Window Hypothesis and MBO Framework. Schematic illustrating the paradigm shift from peak-centric ablation to valley-dose preservation. Biological Mechanism: High-dose peaks trigger direct tumor necrosis, while the optimized Dvalley window (typically 2–4 Gy) selectively preserves vascular patency and immune effector niches, fostering a synergistic microenvironment for systemic response. The MBO Chain (bottom): The flowchart maps the translational pathway from Spatial Dose Modulation (Mechanism) to early Surrogate End points (Biomarkers, e.g., K^trans^, TNF-α), ultimately dictating the balance between Local Control and Normal Tissue Toxicity (Outcome). Note: This conceptual framework is synthesized from the evidence aggregated in this review

Synthesis of these data suggests that the absolute Dvalley—rather than peak dose—emerges as a strong candidate modulator of the therapeutic ratio in SFRT. However, the biological mechanisms underpinning this observed association remain to be fully elucidated. The reviewed literature collectively points toward a biologically driven optimal dose window for the valley region, where in tumor ablation at the vertices is balanced by the preservation of vascular function and immune effector niches within the low-dose inter-vertex corridors (Fig. 1). This pattern, identified across available preclinical and emerging clinical data, constitutes a testable hypothesis necessitating prospective validation.

Consequently, we conducted a PRISMA-ScR scoping review [15] of 60 studies (comprising 16 preclinical studies, 26 clinical studies and 18 technical reports) to map the current evidence landscape. The objectives of this synthesis were to map the evidence across four interconnected domains:

i. Mapping Dvalley–outcome relationships across heterogeneous techni ques to delineate the range of modality-specific thresholds reported (Level 1);
ii. Identifying spatial dose gradient associations with specific biological cascades (e.g., vascular remodeling and immune effector activation) via a candidate Mechanism- Biomarker-Outcome (MBO) chain (Level 2);
iii. Cataloguing early surrogate endpoints under investigation, including functional imaging (PET/SUVmax) and circulating cytokines (TNF-α), capable of estimating treatment durability (Level 3); and.
iv. Synthesizing these findings into an evidence-informed organizational structure to inform future prospective trial designs.

By consolidating the evidence around valley-dose preservation, this review provides a systematic map of the current landscape and offers a hypothesis-generating structure to guide the next generation of spatially fractionated prescriptions.

## Methods

### Study design and framework

This scoping review was conducted in accordance with the PRISMA-ScR guidelines [15]. The study followed the methodological framework originally proposed by Arksey and O’Malley [16] and further refined by Levac et al. [17], comprising six stages: (1) identifying the research question; (2) identifying relevant studies; (3) study selection; (4) charting the data; (5) collating, summarizing, and reporting the results; and (6) expert consultation (optional). Given the heterogeneity of SFRT evidence across preclinical, technical, and clinical domains, this methodology was chosen to map the existing landscape and to examine the role of valley dose across diverse treatment modalities.

### Search strategy and information sources

A systematic search was performed across four electronic databases: PubMed/MEDLINE, Embase, Scopus, and Web of Science, covering the period from January 1, 2005, to July1, 2025. The last search was executed on April 30, 2026. The search string combined MeSH terms and free-text keywords for spatial fractionation (e.g., “spatially fractionated radiation therapy”, “SFRT”, “GRID”, “LATTICE”, “minibeam”, “microbeam”) and dosimetric parameters (“valley dose”, “peak dose”, “peak-to-valley dose ratio”, “PVDR”). The full search strategy is provided in Appendix Table S1. To ensure evidential saturation, reference lists of included studies and relevant reviews were manually screened (backward citation searching).

### Eligibility criteria

To resolve the prevalent terminological conflation between “spatially fractionated” and standard “single-fraction” radiotherapy [18], strict criteria were applied.

#### Inclusion criteria

(i) peer-reviewed original research and secondary syntheses (e.g., systematic reviews, meta-analyses) (ii) use of spatial dose modulation (GRID, LATTICE, or minibeam/microbeam) via photons, protons, or heavy ions; (iii) reporting of at least one spatial dosimetric parameter (e.g., Dvalley, PVDR, or D_peak_).

#### Exclusion criteria

(i) standard uniform-dose stereotactic body radiotherapy (SBRT) or radiosurgery (SRS) protocols without intentional spatial dose modulation; (ii) studies lacking clear dosimetric reporting; (iii) conference abstracts, editorials, or non-English publications. No restriction was placed on publication status (e.g., preprints were considered if peer-reviewed).

### Study selection process

**Fig. 2:**
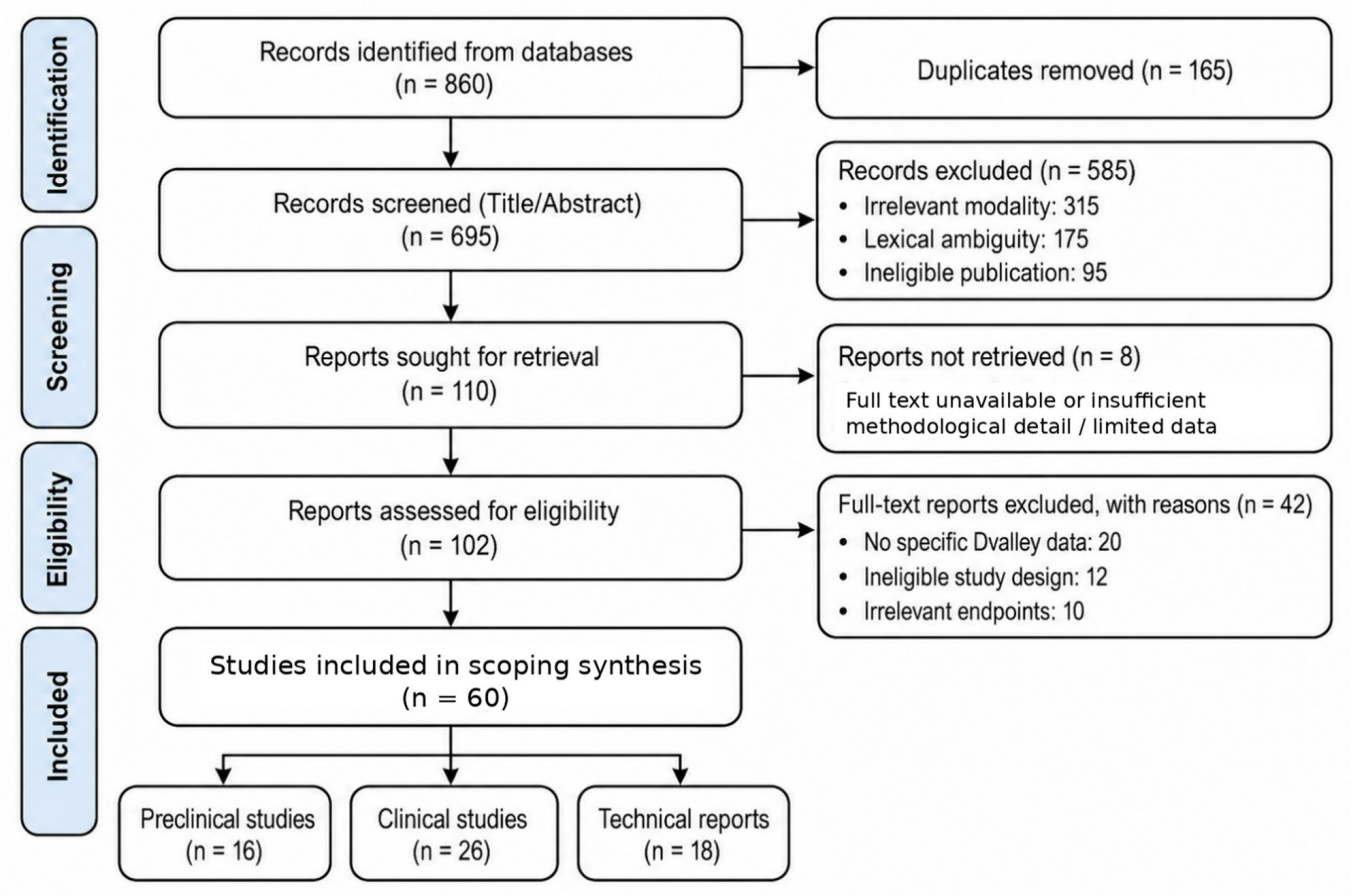
PRISMA-ScR flow diagram of the study selection process

Two reviewers (S.B., C.Z.) independently screened titles and abstracts, followed by full-text review. A pilot calibration exercise was performed on 5% of randomly selected records to ensure consistent application of eligibility criteria (agreement rate: 92%). Discrepancies were resolved by discussion or by a third senior reviewer (Z.H.). The screening process is documented in the PRISMA-ScR flow diagram (Fig. 2). From an initial 860 records, 165 duplicates were removed. After screening 695 records at the title/abstract level and assessing 102 full-text reports, 60 studies were ultimately included, comprising 16 preclinical studies, 26 clinical studies, and 18 technical/ dosimetric reports.

### Data charting and methodological rigor

Data were extracted using a charting form that was iteratively refined through a pilot assessment of five representative studies. Two reviewers (S.B., C.Z.) independently mapped variables into three thematic modules to support the proposed translational frameworks: **(1) Geometric-Dosimetric metric**s (D_peak_, D_valley_, PVDR, and tumor volume/depth); **(2) Mechanistic signatures within the MBO framework** (e.g., CD8 + infiltration, HMGB1/ATP levels); and **(3) Prognostic and Clinical Endpoints**: encompassing early functional imaging surrogates (e.g., PET SUVmax and DCE-MRI K^trans^) alongside definitive clinical outcomes, including Objective Response Rate (ORR), local control, and treatment-related toxicity.

While methodological appraisal is optional for scoping reviews, we performed a targeted quality assessment to weigh the evidence underpinning our D_valley_ -driven models. We adapted the JBI Critical Appraisal tools to address technical nuances specific to SFRT, such as the precision of small-field dosimetry and the rigor of biological replication. This process served to identify evidentiary gaps rather than as a basis for study exclusion. Detailed criteria and the variable list are provided in Appendix Table S2.

### Synthesis of results

Given the high heterogeneity in tumor types, SFRT techniques, and outcome definitions, a quantitative meta-analysis was precluded. Instead, we performed a qualitative thematic synthesis [19]. Extracted data were categorized by dosimetric parameter. Given its repeated prominence in the screened literature, special attention was directed to D_valley_ as a parameter of interest. For studies with incomplete or missing outcome metrics, only available information was reported. The synthesis is presented narratively, supported by summary tables and figures.

## Results

### Evidence mapping and study characteristics

The systematic search (January 2005 – February 2026) identified 860 records, of which 60 landmark studies met the stringent inclusion criteria for final synthesis, which are listed in Appendix Table 3. This curated dataset reflects a progressive shift in the SFRT literature from 2D-GRID empiricism toward D_valley_-centric 3D optimization. Although the search spanned over two decades, the evidence base exhibits a purposeful recency bias, with approximately 80% of the included studies published after 2015. This This temporal clustering reflects the technological maturation of the field: while pioneering works [6–8] established the foundational dosing principles for the valley region, contemporary 3D-LATTICE and Microbeam (MBRT) modalities (2017–2026) [20–25], as illustrated by advanced in vitro models in Bustillo et al. [21] and dose-heterogeneity studies in Potiron et al. [22], with supporting preclinical geometries evaluated in Tsubouchi et al. [23], have transitioned toward high-fidelity D_valley_ optimization as a prerequisite for reporting clinical outcomes [11, 12]. In alignment with this shift toward clinical integration, this synthesis excludes purely dosimetric reports lacking biological correlates, thereby ensuring that the proposed framework is built upon the most robust, outcome-linked evidence currently available.

The resulting dataset comprises a strategic blend of primary preclinical/ clinical trials and high-level secondary syntheses, such as the Ghaznavi et al. (2025) review on emerging strategies in spatial fractionation, ultra-high dose rates, and nanoparticles [24]. These systematic reviews were integrated as “aggregated benchmarks” to validate the robustness of the D_valley_ thresholds identified across individual institutional registries.

The synthesis of the included studies revealed a natural clustering of evidence across three interconnected domains. We present these below not as a validated model, but as an evidence-informed heuristic to organize the current literature and highlight candidate relationships for future prospective testing. Based on this curated evidence, the results are synthesized into a three-level translational framework:

Level 1: Dosimetric Benchmarks – Histology-specific D_valley_ targets.

Level 2: Mechanistic Pathways – D_valley_-dependent biological cascades.

Level 3: Exploratory Surrogates – Short-term metrics for long-term prognosis.

### Level 1: Dosimetric decision-making–tumor-and modality-specific dvalley thresholds

A primary obstacle to the clinical translation of SFRT has been the absence of evidence-based, histology-specific dose guidelines [18, 25–27]. Historically, prescription paradigms focused on D_peak_and the PVDR, operating under the assumption that higher peaks achieve tumor ablation while elevated PVDR spares normal tissues [26, 28, 29]. However, a recurring theme across the 60 curated studies was the strong correlation of D_valley_ with therapeutic outcomes, shifting the focus from “peak intensity” to “inter-track connectivity” [1, 12, 27, 30].

The correlation between D_valley_ and tumor response is increasingly validated across modalities. In preclinical murine models, D_valley_ demonstrated a high correlation with tumor growth delay (R2 > 0.70), whereas D_peak_ often showed non-significant correlation in the same cohorts [11, 12, 31]. Clinical translation mirrors this: in a key study of bulky cervical cancer treated with photon LATTICE (LRT), D_valley_ ≥2 Gy was associated with high local control rates (~ 80%), a significant improvement over historical bulky disease outcomes [13]. For unresectable sarcomas, SFRT (typically delivered with low-to-intermedia te valley doses) has yielded high clinical and radiographic response rates (e.g., ~ 90% stable or partial response) rather than the lower rates historically seen with conventional fractionation alone [32]. In proton minibeam radiotherapy (pMBRT), maintaining a D_valley_ ceiling (e.g., ~ 10 Gy in brain tissue) has been shown to preserve cognitive function and prevent radionecrosis, even with peak doses exceeding 100 Gy [14, 33, 34]. As synthesized from 23 key studies [13, 35–56], D_valley_ thresholds are highly histology-dependent, functioning as both a trigger for efficacy and a ‘safety ceiling’ for normal tissue preservation (Table 1). For intracranial lesions, pMBRT typically utilizes a conservative D_valley_ of ~ 10 Gy paired with a high PVDR (5–8:1) to prevent symptomatic radionecrosis in the CNS [38, 54]. Conversely, pelvic tumors (e.g., GYN or bulky sarcomas) demonstrate a higher tolerance, where a D_valley_ ≥2–3 Gy (peripheral/valley) can be achieved with moderate PVDR (2–4:1) to maximize tumor debulking without exceeding OAR constraints [38, 42, 56]. These benchmarks represent a shift toward precision SFRT, serving as hypothesis- generating targets for upcoming prospective trials [57–60].

**Table 1:** Evidence matrix of D_valley_ benchmarks and clinical out comes (2015– 2026)

Study (Ref)	Year	Tumor Type	Modality	D_valley_ Specifics	Key Clinical / Biological Outcomes
Amendola et al. [42]	2019	NSCLC (Bulky)	LATTICE	Periphery ~ 3 Gy/fx	Mean tumor volume reduction 42%; safe and effective with limited OAR toxicity
Amendola et al. [58]	2020	Cervical Cancer	LATTICE	Periphery ~ 3 Gy/fx	~ 89% complete metabolic response; high local control superior to historical controls
Lu et al. [52]	2024	Advanced NSCLC	SFRT + IO	Low-valley preservation (~ 2–5 Gy range)	Significant abscopal effect; enhanced immune synergy
Sheikh et al. [39]	2025	Various (Bulky)	Proton LRT	Valley dose control	First clinical report of proton LATTICE; safe dose escalation via valley control
Li et al. [47]	2025	Meta-analysis	GRID/LRT	Dose mapping	Confirmed safety profile for large-volume tumors (multi-center pooled data)
Studer et al. [57]	2025	Large Tumors (Palliative)	LATTICE	Threshold-based optimization	Diagnosis-related outcomes; D_valley_ optimization highly correlated with palliation success
Majercakova et al. [53]	2025	Sarcoma (Bulky)	LATTICE	Periphery low-to-intermediate valley dose	67% stable disease; 100% symptom relief (33% conversion to resectable)
Wang et al. [56]	2026	Recurrent GYN (Bulky Cervical)	LATTICE	Tailored low valley dose	Multi-institutional review support; enables re-irradiation of previously treated sites with synergistic radical treatment
Prezado et al. [54]	2025	Brain (Re-irradiation)	Proton MBRT	~ 10 ± 1 Gy	Safety endpoint confirmed; no significant radionecrosis, cognitive function preserved

### Level 2: Mechanism-biomarker pathways – linking valley dose- dependent biology to clinical endpoints

**Fig. 3:**
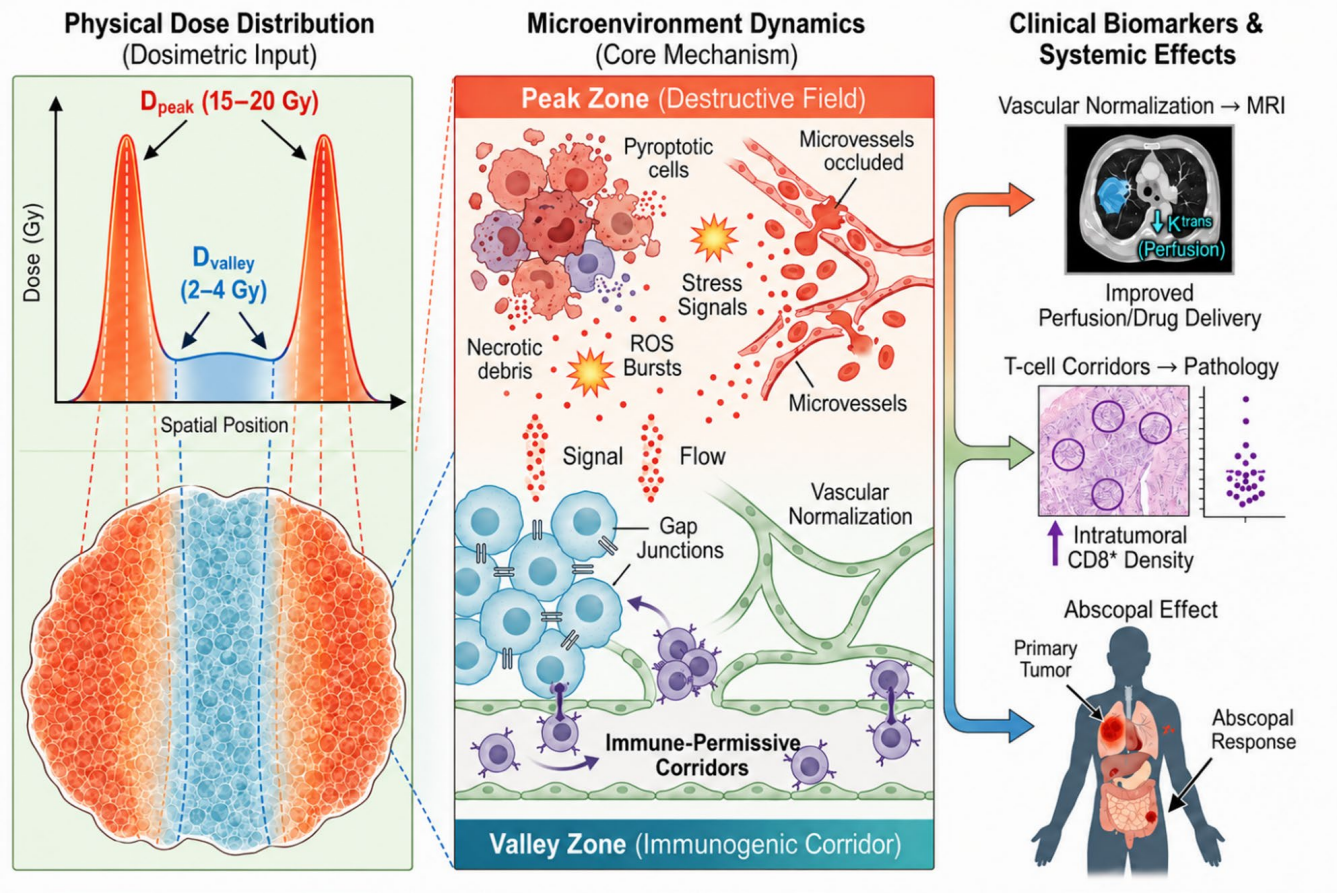
The translational landscape of D_valley_-dependent bio-mechanisms. The The framework illustrates how spatial dose modulation (D_peak_ vs. D_valley)_ dictates distinct biological fates. While peak regions induce acute vascular collapse and necrosis, the 2–4 Gy valley dose maintains “immune-permissive corridors” and induces vascular normalization [52, 54, 61, 62]. These mechanisms are quantifiable via clinical biomarkers: Ktrans for perfusion efficiency and CD8 + density for immune activation [45, 52], collectively driving the systemic abscopal response [38, 48, 52]

Preclinical evidence suggests that key biological cascades associated with the therapeutic advantage of SFRT exhibit D_valley_-dependence [4, 42, 52]. This section organizes these mechanisms into a translational framework connecting spatial dose modulation to measurable clinical biomarkers [15, 29, 38, 47]. The integrated landscape of these dose-dependent biological cascades and their corresponding clinical biomarkers is illustrated in Fig. 3.

#### Vascular remodeling and perfusion efficiency

In rat fibrosarcoma models, SFRT with D_valley_ in the 2–4 Gy range has been shown to drive tumor response primarily through valley-dose mechanisms, with effects more pronounced than uniform irradiation at equivalent mean doses [11, 35]. Unlike the complete vascular obliteration seen in high-dose regions, this “moderate” valley dose contributes to a vascular normalization phenotype, potentially facilitating systemic drug penetration [14, 48, 61].

##### Clinical surrogate

A candidate clinical biomarker for this effect is the volume transfer constant (Ktrans) derived from DCE-MRI. Preliminary evidence from lung cancer radiotherapy studies indicates that a post-RT reduction in Ktrans correlates with improved tumor response rates, although this link requires multi-center prospective validation [48, 62–64].

#### Immune microenvironment preservation

The “low-dose corridors” within the valley regions are hypothesized to safeguard the immune microenvironment [48, 52].

##### Mechanism

In murine mammary carcinoma models, SFRT with D_valley_ levels around 3.7 Gy (combined with anti-PD-L1) led to a significant increase in intratumoral CD8 + T-cell infiltration and superior abscopal effect compared to conventional uniform irradiation [65]. Clinically, preliminary reports from cohorts combining SFRT with anti-PD-1 therapy (e.g., for advanced NSCLC) have shown promising systemic responses (Fig. 3, right panels).

##### Clinical translation

Tubin et al. reported high bystander and abscopal responses (e.g.,~52% abscopal rate) in patients treated with SFRT/SBRT -PATHY directed at the tumor center, suggesting that D_valley_ dose-shaping effectively preserves the immune microenvironment [66]. While post-RT CD8 + T-cell density has been identified as a favorable prognostic indicator in preclinical models, its role as a definitive clinical biomarker for SFRT-immuno therapy synergy is currently being validated in ongoing prospective trials [38].

##### Clinical surrogate

A candidate clinical biomarker for this effect is the volume transfer constant (K^trans^) derived from DCE-MRI. Preliminary evidence from lung cancer studies indicates that a post-RT reduction in K^trans^ correlates with improved tumor response rates, although this link requires multi-center prospective validation [62, 63].

#### Bystander signaling and the FLASH-SFRT frontier

Preclinical models using A549 and other lung cancer cell lines suggest that the therapeutic advantage of SFRT is partially mediated by potent bystander effects. Unlike the uniform cell kill of conventional RT, the steep dose gradients in SFRT trigger the release of Reactive Oxygen Species (ROS) and facilitate intercellular communication via gap junctions, inducing DNA double-strand breaks in adjacent, non-irradiated cells [67, 68].

Clinically, while systemic pro-inflammatory markers like TNF-α and interleukin-6 (IL-6) have been proposed as surrogates to monitor these non-targeted effects, their direct correlation with long-term local control remains a subject of active investigation [69]. Furthermore, the emerging frontier of FLASH-SFRT (integrating ultra-high-dose-rate delivery) represents a paradigm shift. By exploiting the differential oxygen depletion kinetics between peaks and valleys, FLASH-SFRT is hypothesized to amplify the therapeutic window—maximizing bystander-mediated tumor destruction while significantly attenuating normal tissue toxicity [70, 71].

To facilitate clinical translation, the intricate interplay between these D_valley_-dependent biological cascades, their validated preclinical dose ranges, and their corresponding clinical surrogates is synthesized in Table 2.

**Table 2:** Mechanistic landscape: linking D_valley_ to biological cascades and biomarkers

Biological Dimension	Mechanism of Action	Key Preclinical Evidence (Ref)	Critical D_valley_ Range	Candidate Clinical Biomarker
Vascular Dynamics	Vascular normalization; reduction in MVD and interstitial pressure	Ahmed et al. (2024); Rivera et al. (2020).[11, 48]	2–4 Gy	DCE-MRI (Ktrans); post-RT ΔKtrans reduction
Bystander Signaling	Intercellular communication via Gap Junctions; ROS-mediated DNA damage	Asur et al. (2012); Autsavapromporn et al.(2023)[67, 68]	Steep Gradients	Cytokine Profiling (e.g., TNF-α, IL-6, HMGB1)
Metabolic Shift	Reversal of hypoxia-induced resistance; metabolic reprogramming	Prezado et al. (2024); Fang et al. (2025) [14, 61]	≥ 2 Gy	PET Imaging (18 F-MISO); Lactate levels
FLASH Synergy	Ultra-high-dose-rate sparing of normal stem cells in valley regions	Schneider et al. (2022);Diffenderfer et al. (2020) [70, 71]	Dose-rate dependent	ΔpO2 (Real-time oxygen tension)

### Level 3: Exploratory surrogate endpoints – short-term metrics to project long-term local control

To bridge the gap between D_valley_-based dose prescription and long-term clinical validation, the reviewed literature identifies several exploratory surrogate endpoints under evaluation in institutional cohorts. A significant limitation of the current SFRT literature remains the scarcity of long-term (≥ 5-year) prospective data, with most LATTICE and GRID studies reporting 12–24 month follow-up [47, 57]. Consequently, short-term functional imaging and circulating markers are being investigated not as definitive predictors, but as hypothesis-generating surrogates for treatment durability [42, 69, 72].

**Imaging-based surrogates: The Role of PET-Response** In institutional series of bulky NSCLC and sarcomas, early metabolic response has emerged as a promising but preliminary indicator. Amendola et al. (2019) observed that rapid tumor volume reduction (mean 42%) and complete metabolic responses were characteristic of patients achieving sustained local control [42]. While specific long-term LC rates remain to be validated in multi-center settings, Li et al. (2025) meta-analysis confirms that SFRT achieves a pooled objective response rate (ORR) of approximately 79% across various histologies [47]. Ferini et al. (2022) further demonstrated metabolism-guided LATTICE yielding high early metabolic CR rates in bulky tumors [72]. Thus, early metabolic regression serves as a high-confidence surrogate for the “debulking” efficacy of the D_peak_ and the peripheral tumor control driven by D_valley_ [14, 42].

**Circulating biomarkers: Cytokine dynamics** Beyond imaging, systemic inflammatory signaling—particularly via cytokines such as TNF-α—has emerged as a potential physiological mirror of the D_valley_-mediated bystander effect. Historical studies in GRID therapy have consistently noted significant post-irradiation fluctuations in serum TNF-α, which are hypothesized to trigger systemic anti-tumor immunity [69]. Current evidence indicates that these cytokines may play a bifurcated prognostic role: while some reports suggest that sustained suppression of pro-inflammatory markers correlates with superior local control by reflecting reduced tumor burden, others indicate that persistence of systemic elevation may be a prerequisite for robust abscopal signals [69]. This dual-directional significance remains a major barrier to establishing standardized prognostic thresholds. Furthermore, the lack of standardized assays and the inherent heterogeneity between histologies highlight the urgent need for multi-center validation [14, 49, 69].

**Fig. 4:**
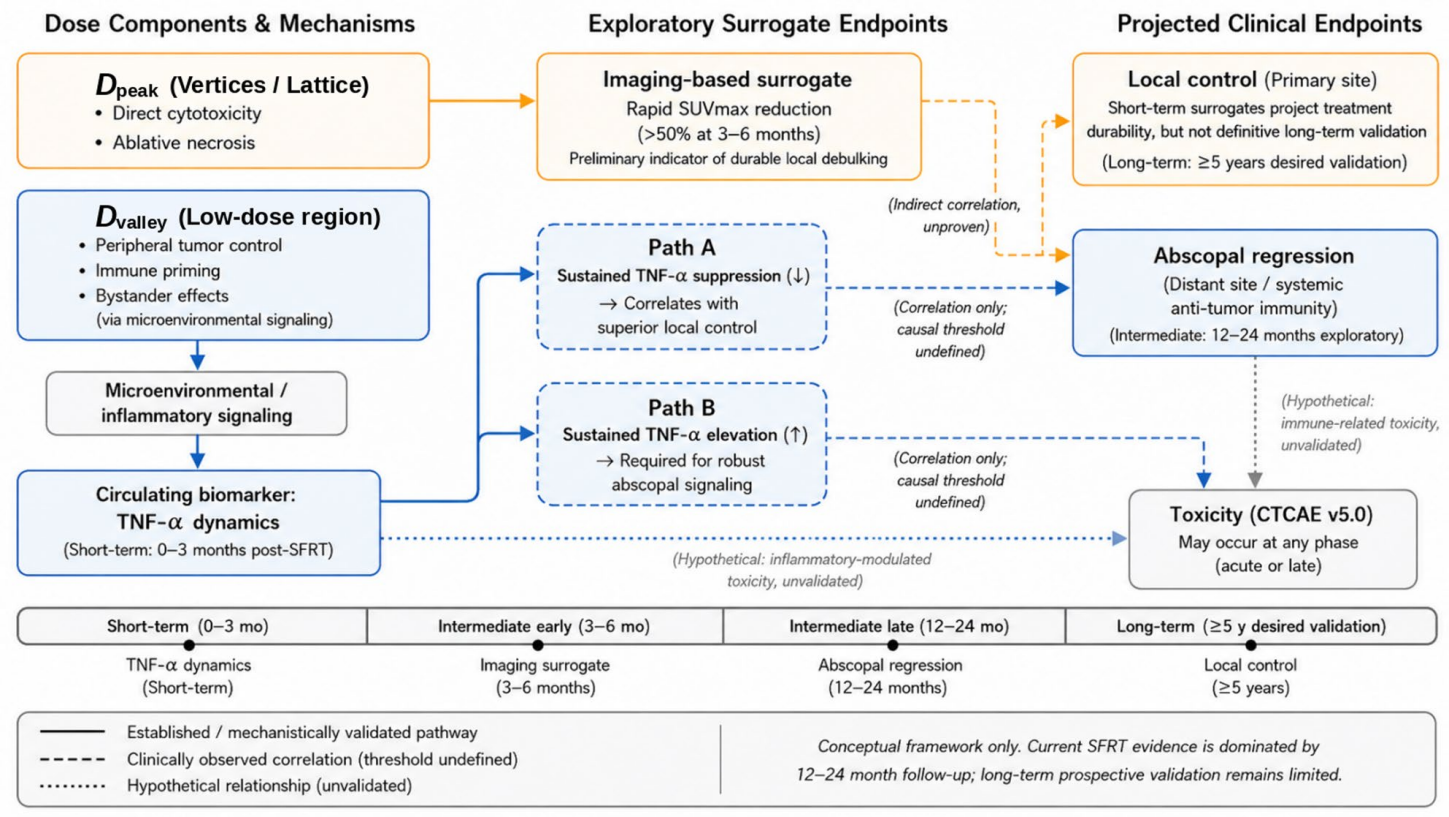
Evidence-informed conceptual structure: Hypothesized relation ships from dose components to clinical validation emerging from the reviewed literature. SFRT dose distributions (D_peak_, D_valley_) trigger distinct biological mechanisms (cytotoxicity, vascular normalization, immune priming, bystander effects). These can be monitored via short-term surrogates: early metabolic response (≥ 50% SUVmax reduction or rapid tumor volume reduction) and TNF-α dynamics. Surrogates project long-term outcomes: local control, abscopal regression, and toxicity (CTCAE v5.0, acute/late). Solid lines = pathways supported by included studies; dashed lines = hypothetical relationships. Notes: Sustained cytokine suppression (Path A) is proposed as a marker for local stability; systemic elevation (Path B) is hypothesized as a prerequisite for abscopal effects. Toxicity may occur at any phase. References [14, 42, 47, 57, 69, 72] provide supporting data. Abbreviations: SFRT, spatially fractionated radiation therapy; SUV, standardized uptake value; LC, local control; CTCAE, Common Terminology Criteria for Adverse Events

To synthesize these diverse biological observations into a cohesive translational model, we propose a D_valley_-driven conceptual framework. This framework is designed to bridge the current evidence gap by utilizing short-term functional metrics as surrogate benchmarks for long-term clinical durability. As illustrated in Fig. 4, the model delineates a three-stage trajectory:

#### Dose-to-mechanism coupling

It distinguishes between the ablative cytotoxicity driven by D_peak_ and the microenvironmental “priming” (vascular normalization, immune priming, and bystander effects) orchestrated by the D_valley_ region.

#### Surrogate-based projection

A key feature of this framework is its predictive layer, where early metabolic shifts and cytokine dynamics serve as physiological mirrors of treatment efficacy.

#### Clinical endpoint validation

By linking these early signals to long-term LC, systemic abscopal responses, and toxicity (CTCAE v5.0, acute/late), the framework provides a roadmap for future multi-center trials to validate whether early inflammatory signatures can reliably anticipate treatment outcomes and potential immune-related toxicities.

This conceptual shift from purely physical dose-reporting to a biologically-guided “ surrogate-to-endpoint” projection is essential for establishing standardized, clinically actionable benchmarks in SFRT.

## Discussion

The present review identifies a strong and recurrent pattern in spatially fractionated dosimetry: the prominent association of D_valley_ with therapeutic outcomes [54, 73]. While the historical focus of GRID therapy resided in the ablative capacity of the peaks [7], our data synthesis suggests that the clinical success of SFRT may be more dependent on inter-vertex preservation than previously recognized [74]. We propose a three-stage translational trajectory (Fig. 4) that moves beyond empirical observations toward a predictable, biology-guided “surrogate-to-endpoint” projection. This framework explicitly bridges the current evidence gap—namely, the scarcity of five-year prospective data—by utilizing early functional metrics, such as metabolic kinetics [75] and bifurcated cytokine dynamics (the “TNF-α paradox”) [76], as mechanistic mirrors of long-term clinical durability.

Specifically, by anchoring D_valley_ to a hypothesized cGAS-STING-mediated immune response [77, 78] and vascular “conduits” [54], we offer a potential mechanistic explanation for why identical peak doses can yield disparate systemic outcomes across different spatial geometries [36]. This hypothesis remains testable and requires dedicated validation. The proposed shift would necessitate a re-calibration of planning priorities, such that D_valley_ is no longer treated as a passive byproduct but as a primary optimization constraint [49, 73, 74].

### Comparison with previous reviews

Prior SFRT reviews have largely focused on technical parameters (e.g., PVDR ranges, collimator design) or have listed efficacy data from individual studies without integrating them into a coherent translational framework [1, 24]. Some have described preclinical mechanisms (vascular modulation, bystander effects) but offered no path to validate these in humans [13, 75]. To our knowledge, no previous review has systematically examined valley dose as a unifying dosimetric anchor across different SFRT techniques. While the evidence remains preliminary, this synthesis provides a testable hypothesis—that valley dose is strongly associated with outcome—and a structured evidence map that researchers can use to prioritize which thresholds, biomarkers, and surrogates to validate in future prospective trials [38].

### Limitations

While this review provides a systematic map of the SFRT landscape, several inherent limitations must be considered.

Primarily, the current evidence base is characterized by a conspicuous deficit of high-level clinical data [14, 38]; most included studies are small, single-center retrospective cohorts, with randomized controlled trials representing only a minor fraction of the literature. This scarcity, compounded by significant heterogeneity in tumor histology and SFRT delivery techniques, precluded a formal quantitative meta- analysis [1].

Furthermore, the proposed D_valley_thresholds and the associated mechanism- biomarker chain (e.g., CD8 + T-cell dynamics) remain largely translational. Many of these metrics are extrapolated from murine models or treatment planning simulations rather than prospective human cohorts, necessitating cautious interpretation [73]. Consequently, It must be emphasized that the “TNFTNF-α paradox” and the bifurcated trajectories (Path A and Path B) illustrated in our framework (Fig. 4) should be viewed as hypothesis-generating rather than definitive.

Finally, the surrogate endpoint layer lacks long-term validation. The absence of 5-year survival data and standardized predictive metrics (e.g., AUC, calibration) in the current SFRT literature limits the immediate clinical utility of these surrogates. Despite our efforts to isolate SFRT-specific effects, the potential for residual confounding from conventional single-fraction radiotherapy paradigms cannot be entirely dismissed. These gaps underscore the urgent need for standardized multicenter protocols to transition SFRT from an exploratory technique to a precision-guided clinical standard [1, 14].

### Future perspectives: From heuristics to precision spatially fractionated radiotherapy

The evidence mapped in this review supports the further exploration of a “Valley-Dose Window” hypothesis, which may represent an important transition in SFRT dosimetry. This working model, distilled from the reviewed studies, posits that the therapeutic ratio is not merely a function of ablative peaks, but is fundamentally governed by a dosimetric “Goldilocks zone” within the valley regions. We identify four critical pillars supporting this paradigm: (i) the requirement of a minimum D_valley_ to prevent marginal tumor recurrence; (ii) a biological ceiling above which the “Sparing Effect” on microvasculature and immune niches is lost; (iii) the dose‑dependent activation of the cGAS‑STING pathway; and (iv) the preservation of spatial heterogeneity as an enabling condition for the bystander/abscopal effect. While the first pillar is anchored in emerging clinical observations, the latter three remain hypothesis‑generating, requiring a transition from retrospective “snapshots” to prospective, biology‑driven validation.

#### Refining the radiobiological kinetics of the valley zone

A priority for the next generation of SFRT research is to refine current histology‑agnostic empirical thresholds toward precise dose‑response kernels. Most existing data derive from small, heterogeneous cohorts (*n* < 50) that do not adequately capture the variability in stromal resilience across different tumor microenvironments [1, 18].

A key priority is to deconvolve the interplay between D_valley_ and the PVDR. The reviewed data suggest the possibility that a high PVDR is not an intrinsic biological requirement but a dosimetric proxy for maintaining D_valley_ below a critical toxicity threshold. Moving forward, “mean valley dose” metrics could be complemented by spatial dose‑volume effects (SDVE). By leveraging machine learning to map voxel‑level dosimetry, we can begin to quantify the functional connectivity of spared tissue corridors—the essential “bio‑conduits” through which immune trafficking and systemic signaling are mediated [73, 79].

Beyond voxel-level dosimetry, future research must validate the bifurcated signaling of TNF-α as a dynamic biomarker. As conceptualized in our model, the distinction between sustained suppression (correlating with local stability) and systemic elevation (required for abscopal induction) offers a potential ‘biological compass’ for SFRT. Integrating these inflammatory signatures with early SUVmax kinetics (e.g., the ≥ 50% reduction threshold) will be critical for transitioning from static dose-reporting to real-time, adaptive treatment monitoring.

#### Clinical translation and workflow integration

The transition of SFRT from a niche salvage technique to a standard‑of‑care modality is currently limited by the lack of professionalized planning infrastructure. The historical reliance on manual vertex placement contributes to inter‑institutional variability and complicates cross‑study aggregation. We advocate for the development of native, biology‑guided optimization kernels within commercial treatment planning systems (TPS), where D_valley_ constraints are integrated as primary constraints in inverse‑planning objective functions [80].

Simultaneously, the exploratory surrogate endpoints proposed in this review, including early SUVmax kinetics and circulating radiomic signatures, should be validated through multicenter master protocols [81]. A standardized reporting nomenclature (analogous to the AAPM TG‑263 for conventional RT) would facilitate data aggregation across centers. Such harmonization is an important step toward generating the evidence base needed for curative‑intent applications of SFRT [38].

For centers without proton capability, the photon-based techniques included in the framework (e.g., VMAT-GRID for lung tumors, LATTICE radiotherapy for cervical cancer) may serve as practical alternatives; however, optimal thresholds may differ, and local validation against institutional data is advised.

## Conclusion

This scoping review of 60 studies identifies a consistent association between D_valley_ and both tumor control and normal tissue sparing across major SFRT modalities. Synthesizing the current evidence, we delineate a valley-driven, biology-guided translational framework—presented as an evidence-informed heuristic—that links D_valley_ thresholds to downstream vascular and immune modulation, and further to early metabolic surrogates as candidate correlates of long-term efficacy. However, considerable heterogeneity in the literature renders these threshold and biomarker associations hypothesis-generating. Prospective, multicenter dose-finding trials that anchor planning to D_valley_ are urgently needed to validate these relationships and advance SFRT from an exploratory salvage option to a precision-guided standard.

## Supplementary Information

Below is the link to the electronic supplementary material.

Supplementary Material 1

## Data Availability

No datasets were generated or analysed during the current study.

## Abbreviations

SFRT: Spatially fractionated radiation therapy
D_valley_: Valley dose
D_peak_: Peak dose
PVDR: Peak-to-valley dose ratio
GRID: Grid therapy
LRT: LATTICE radiotherapy
MRT: Microbeam radiotherapy
MBRT: Minibeam radiotherapy
pMBRT: Proton minibeam radiotherapy
PRISMA-ScR: Preferred Reporting Items for Systematic reviews and Meta-Analyses extension for Scoping Reviews
JBI: Joanna Briggs Institute
OAR: Organ at risk
TCP: Tumor control probability
NTCP: Normal tissue complication probability
MBO: Mechanism-biomarker-outcome
DCE-MRI: Dynamic contrast-enhanced magnetic resonance imaging
Ktrans: Volume transfer constant
SUVmax: Maximum standardized uptake value
PET: Positron emission tomography
ORR: Objective response rate
LC: Local control
CTCAE: Common Terminology Criteria for Adverse Events
NSCLC: Non-small-cell lung cancer
VMAT: Volumetric modulated arc therapy
SBRT: Stereotactic body radiotherapy
SRS: Stereotactic radiosurgery
ROS: Reactive oxygen species
HMGB1: High-mobility group box 1
TNF-α: Tumor necrosis factor-alpha
PD-1: Programmed cell death protein 1
PD-L1: Programmed death-ligand 1
WHO ICTRP: World Health Organization International Clinical Trials Registry Platform
ChiCTR: Chinese Clinical Trial Registry

## References

[1] Billena C, Khan AJ. A current review of spatial fractionation: back to the future? Int J Radiat Oncol Biol Phys. 2019;104(1):177–87. 10.1016/j.ijrobp.2019.01.073. 30684666 10.1016/j.ijrobp.2019.01.073 PMC7443362

[2] Lievens Y, Guckenberger M, Gomez D, et al. Defining oligometastatic disease from a radiation oncology perspective: an ESTRO-ASTRO consensus document. Radiother Oncol. 2020;148:157–66. 10.1016/j.radonc.2020.04.003. 32388150 10.1016/j.radonc.2020.04.003

[3] Castorina P, Castiglione F, Ferini G, Forte S, Martorana E. Computational approach for spatially fractionated radiation therapy (SFRT) and immunological response in precision radiation therapy. J Pers Med. 2024;14(4):436. 10.3390/jpm14040436. 38673063 10.3390/jpm14040436 PMC11051362

[4] Kanagavelu S, Gupta S, Wu X, et al. In vivo effects of lattice radiation therapy on local and distant lung cancer: potential role of immunomodulation. Radiat Res. 2014;182(2):149–62. 10.1667/RR3819.1. 25036982 10.1667/RR3819.1 PMC7670883

[5] Kelly DA. Treatment of multiple brain metastases with a divide-and- conquer spatial fractionation radiosurgery approach. Med Hypotheses. 2014;83(4):425–8. 10.1016/j.mehy.2014.04.024. 25175716 10.1016/j.mehy.2014.04.024

[6] Prezado Y, Fois GR. Proton-minibeam radiation therapy: a proof of concept. Med Phys. 2013;40(3):031712. 10.1118/1.4791648. 23464307 10.1118/1.4791648

[7] Mohiuddin M, Fujita M, Regine WF, Megooni AS, Ibbott GS, Ahmed MM. High-dose spatially-fractionated radiation (GRID): a new paradigm in the management of advanced cancers. Int J Radiat Oncol Biol Phys. 1999;45(3):721–7. 10.1016/s0360-3016(99)00170-4. 10524428 10.1016/s0360-3016(99)00170-4

[8] Prezado Y, Jouvion G, Hardy D, et al. Proton minibeam radiation therapy spares normal rat brain: long-term clinical, radiological and histopathological analysis. Sci Rep. 2017;7(1):14403. 10.1038/s41598-017-14786-y. 29089533 10.1038/s41598-017-14786-y PMC5663851

[9] Das IJ, Khan AU, Dogan SK, Longo M. Grid/lattice therapy: consideration of small field dosimetry. Br J Radiol. 2024;97(1158):1088–98. 10.1093/bjr/tqae060. 38552328 10.1093/bjr/tqae060 PMC11135801

[10] Duriseti S, Kavanaugh JA, Szymanski J, et al. LITE SABR M1: a phase I trial of lattice stereotactic body radiotherapy for large tumors. Radiother Oncol. 2022;167:317–22. 10.1016/j.radonc.2021.11.023. 34875286 10.1016/j.radonc.2021.11.023

[11] Rivera JN, Kierski TM, Kasoji SK, et al. Conventional dose rate spatially- fractionated radiation therapy treatment response and its association with dosimetric parameters—a preclinical study in a Fischer 344 rat model. PLoS ONE. 2020;15(6):e0229053. 10.1371/journal. pone. 022 9053. 32569277 10.1371/journal.pone.0229053 PMC7307781

[12] Garcia DA, Fazzari JM, Hlushchuk R, et al. Minibeam radiation therapy valley dose determines tolerance to acute and late effects in the mouse oral cavity. Int J Radiat Oncol Biol Phys. 2025;123(1):262–9. 10.1016/j. ijrobp.2025.03.016. 40097117 10.1016/j.ijrobp.2025.03.016

[13] Amendola BE, Perez NC, Mayr NA, Wu X, Amendola M. Spatially fractionated radiation therapy using lattice radiation in far-advanced bulky cervical cancer: a clinical and molecular imaging and outcome study. Radiat Res. 2020;194(6):724–36. 10.1667/RADE-20-00038.1. 32853384 10.1667/RADE-20-00038.1

[14] Prezado Y. Spatially fractionated radiation therapy: a critical review on current status of clinical and preclinical studies and knowledge gaps. Phys Med Biol. 2024;69(10):10TR02. 10.1088/1361-6560/ad4192. 10.1088/1361-6560/ad4192 38648789

[15] Tricco AC, Lillie E, Zarin W, et al. PRISMA extension for scoping reviews (PRISMA-ScR): checklist and explanation. Ann Intern Med. 2018;169(7):467–73. 10.7326/M18-0850. 30178033 10.7326/M18-0850

[16] Arksey H, O’Malley L. Scoping studies: towards a methodological framework. Int J Soc Res Methodol. 2005;8(1):19–32. 10.1080/1364557032000.

[17] Levac D, Colquhoun H, O’Brien KK. Scoping studies: advancing the methodology. Implement Sci. 2010;5:69. 10.1186/1748-5908-5-69. 20854677 10.1186/1748-5908-5-69 PMC2954944

[18] Giap F, Mohiuddin MM, Wu X, et al. Spatially fractionated radiation therapy: history, present, and future. Int J Radiat Oncol Biol Phys. 2023;117(2):304–24. 10.1016/j.ijrobp.2023.05.020.

[19] Popay J, Roberts H, Sowden A, et al. Guidance on the conduct of narrative synthesis in systematic reviews: a product from the ESRC methods programme.

[20] Dilmanian FA, et al. Merging orthovoltage X-ray minibeams spare the normal brain of rats while still destroying the glioma. Sci Rep. 2019;9:16479. 10.1038/s41598-018-37733-x. 30718607 10.1038/s41598-018-37733-x PMC6362296

[21] Bustillo JPO, Engels EEM, de Rover V, et al. Three-dimensional bioprinted in vitro glioma tumor constructs for synchrotron microbeam radiotherapy dosimetry and biological study using gelatin methacryloyl hydrogel. Sci Rep. 2025;15(1):13868. 10.1038/s41598-025-88793-9. 40263410 10.1038/s41598-025-88793-9 PMC12015499

[22] Potiron S, Iturri L, Juchaux M, et al. The significance of dose hetero geneity on the anti-tumor response of minibeam radiation therapy. Radiother Oncol. 2024;201:110577. 10.1016/j.radonc.2024.110577. 39393469 10.1016/j.radonc.2024.110577

[23] Tsubouchi T, Henry T, Ureba A, et al. Quantitative evaluation of potential irradiation geometries for carbon-ion beam grid therapy. Med Phys. 2018;45(3):1210–21. 10.1002/mp.12749. 29319842 10.1002/mp.12749

[24] Ghaznavi H, Rezaee M, Reynoso F, Darafsheh A. Emerging strategies in radiation therapy: promises and challenges of spatial fractionation, ultra-high dose rates, and nanoparticles. J Phys D Appl Phys. 2025;58(41):413002. 10.1088/1361-6463/ae0e2d. 41089309 10.1088/1361-6463/ae0e2d PMC12516303

[25] Yan W, Khan MK, Wu X, et al. Spatially fractionated radiation therapy: history, present and the future. Clin Transl Radiat Oncol. 2019;20:30–8. 10.1016/j.ctro.2019.10.004. 31768424 10.1016/j.ctro.2019.10.004 PMC6872856

[26] Zhang H, Wu X, Zhang X, et al. Photon GRID radiation therapy: a physics and dosimetry white paper from the Radiosurgery Society (RSS) GRID/LATTICE, microbeam and FLASH radiotherapy working group. Radiat Res. 2020;194(6):665–77. 10.1667/RADE-20-00047.1. 33348375 10.1667/RADE-20-00047.1

[27] Iori F, Cappelli A, D’Angelo E, et al. Lattice radiation therapy in clinical practice: a systematic review. Clin Transl Radiat Oncol. 2022;39:100569. 10.1016/j.ctro. 2022.100569. 36590825 10.1016/j.ctro.2022.100569 PMC9800252

[28] At B, Ramasubramanian V. Comparative analysis of dose gradients and valley doses in pelvic lattice radiotherapy in RapidArc and IMRT. Asian Pac J Cancer Prev. 2024;25(11):4061–6. 10.31557/APJCP.2024.25.11.4061. 39611931 10.31557/APJCP.2024.25.11.4061 PMC11996128

[29] Zhang W, Traneus E, Lin Y, Chen RC, Gao H. A novel treatment planning method via scissor beams for uniform-target-dose proton GRID with peak-valley- dose-ratio optimization. Med Phys. 2024;51(10):7047–56. 10.1002/mp.17307. 39008781 10.1002/mp.17307

[30] Sheikh K, Li H, Wright JL, Yanagihara TK, Halthore A. The peaks and valleys of photon versus proton spatially fractionated radiotherapy. Semin Radiat Oncol. 2024;34(3):292–301. 10.1016/j.semradonc.2024.04.007. 38880538 10.1016/j.semradonc.2024.04.007

[31] Schneider T, Malaise D, Pouzoulet F, Prezado Y. Orthovoltage X-ray minibeam radiation therapy for the treatment of ocular tumours—an in silico evaluation. Cancers (Basel). 2023;15(3):679. 10.3390/cancers15030679. 36765637 10.3390/cancers15030679 PMC9913874

[32] Ahmed SK, Petersen IA, Grams MP, et al. Spatially fractionated radiation therapy in sarcomas: a large single-institution experience. Adv Radiat Oncol. 2024;9(3):101428. 10.1016/j.adro.2023.101428. 38495033 10.1016/j.adro.2023.101401 PMC10943518

[33] Prezado Y, Jouvion G, Guardiola C, et al. Tumor control in RG2 glioma-bearing rats: a comparison between proton minibeam therapy and standard proton therapy. Int J Radiat Oncol Biol Phys. 2019;104(2):266–71. 10.1016/j.ijrobp.2019.01.080. 30703513 10.1016/j.ijrobp.2019.01.080

[34] Bertho A, Ortiz R, Juchaux M, et al. First evaluation of temporal and spatial fractionation in proton minibeam radiation therapy of glioma-bearing rats. Cancers (Basel). 2021;13(19):4865. 10.3390/cancers13194865. 34638352 10.3390/cancers13194865 PMC8507607

[35] Iori F, Botti A, Ciammella P, et al. How a very large sarcomatoid lung cancer was efficiently managed with lattice radiation therapy: a case report. Ann Palliat Med. 2022;11(11):3555–61. 10.21037/apm-22-246. 35871277 10.21037/apm-22-246

[36] Mayr NA, Mohiuddin M, Snider JW, et al. Practice patterns of spatially fractionated radiation therapy: a clinical practice survey. Adv Radiat Oncol. 2023;9(2):101308. 10.1016/j.adro.2023.101308. 38405319 10.1016/j.adro.2023.101308 PMC10885580

[37] Rode I. Clinical and economic significance of massive grid irradiation in the therapy of breast cancer. Acta Chir Acad Sci Hung. 1962;3:viii–xx. 13974601

[38] Li H, Wu X, et al. Overview and recommendations for prospective multi- institutional clinical trials of spatially fractionated radiation therapy (SFRT). Int J Radiat Oncol Biol Phys. 2024;119(3):737–49. 10.1016/j. ijrobp.2023.12.013. 38110104 10.1016/j.ijrobp.2023.12.013 PMC11162930

[39] Sheikh K, Tran A, Li H, et al. Interim analysis of Pro-Grid: a phase 1 proton spatially fractionated radiation therapy trial. Adv Radiat Oncol. 2025;10(9):101857. 10.1016/j.adro.2025.101857. 41625170 10.1016/j.adro.2025.101857 PMC12859408

[40] Ferini G, Valenti V, Viola A, et al. First-ever clinical experience with magnetic resonance-based lattice radiotherapy for treating bulky gynecological tumors. Anticancer Res. 2022;42(9):4641–6. 10.21873/anticanres.15968. 36039437 10.21873/anticanres.15968

[41] Zhang H, Wong K, Olch A, et al. Initial experience of spatially fractionated lattice radiation therapy for palliative treatment of pediatric bulky tumors. Front Oncol. 2025;15:1648847. 10.3389/fonc.2025.1648847. 41220949 10.3389/fonc.2025.1648847 PMC12597734

[42] Amendola BE, Perez NC, Wu X, Amendola MA, Qureshi IZ. Safety and efficacy of lattice radiotherapy in voluminous non-small cell lung cancer. Cureus. 2019;11(3):e4263. 10.7759/cureus.4263. 31139522 10.7759/cureus.4263 PMC6519973

[43] Burns L, Tsai J, Wong P, et al. Spatially fractionated radiotherapy for re-irradiation: feasibility, safety, treatment planning, and outcomes. Clin Transl Radiat Oncol. 2025;56:101049. 10.1016/j.ctro.2025.101049. 41080985 10.1016/j.ctro.2025.101049 PMC12512144

[44] Corvino A, Schneider T, Vu-Bezin J, Loap P, Kirova Y, Prezado Y. Photon mini-GRID therapy for preoperative breast cancer tumor treatment: a treatment plan study. Med Phys. 2025;52(4):2493–506. 10.1002/mp.17634. 39873910 10.1002/mp.17634 PMC11972043

[45] Kersten K, Hu KH, Combes AJ, et al. Spatiotemporal co-dependency between macrophages and exhausted CD8 + T cells in cancer. Cancer Cell. 2022;40(6):624–e6389. 10.1016/j.ccell.2022.05.004. 35623342 10.1016/j.ccell.2022.05.004 PMC9197962

[46] Grams MP, Mateus CQ, Mashayekhi M, et al. Minibeam radiation therapy treatment (MBRT): commissioning and first clinical implementation. Int J Radiat Oncol Biol Phys. 2024;120(5):1423–34. 10.1016/j.ijrobp.2024.06.035. 10.1016/j.ijrobp.2024.06.035 39002850

[47] Li W, Piao M, Zhai L, et al. Effectiveness and safety of lattice radiotherapy in treating large volume tumors: a systematic review and meta-analysis based on single-arm clinical studies. Balkan Med J. 2025;42(4):311–20. 10.4274/balkanmedj.galenos.2025-2-129. 40619801 10.4274/balkanmedj.galenos.2025.2025-2-129 PMC12240221

[48] Ahmed MM, Wu X, Wright J, et al. Optimizing GRID and lattice spatially fractionated radiation therapy: innovative strategies for radioresistant and bulky tumor management. Semin Radiat Oncol. 2024;34(3):310–22. 10.1016/j.semradonc.2024.05.002. 10.1016/j.semradonc.2024.05.002 38880540

[49] Owen D, Harmsen WS, Ahmed SK, et al. Highs and lows of spatially fractionated radiation therapy: dosimetry and clinical outcomes. Pract Radiat Oncol. 2025;15(4):e388–95. 10.1016/j.prro.2024.12.002. 39725127 10.1016/j.prro.2024.12.002

[50] Li Y, Martin-Paulpeter RM, Chung CV, et al. Novel pencil-beam scanning proton lattice radiation therapy for the treatment of bulky liver cancer: dosimetric comparison with VMAT-lattice radiotherapy. Front Oncol. 2026;15:1731259. 10.3389/fonc.2025.1731259. 41716710 10.3389/fonc.2025.1731259 PMC12913141

[51] Qi XS, Ruan D, Lee SP, et al. Dependence of achievable plan quality on treatment technique and planning goal refinement: a head-and-neck intensity modulated radiation therapy application. Int J Radiat Oncol Biol Phys. 2015;91(4):817–24. 10.1016/j.ijrobp.2014.11.037. 25752396 10.1016/j.ijrobp.2014.11.037

[52] Lu Q, et al. Combining spatially fractionated radiation therapy (SFRT) and immunotherapy opens new rays of hope for enhancing therapeutic ratio. Clin Transl Radiat Oncol. 2024;44:100557. 10.1016/j.ctro.2023.100557. 10.1016/j.ctro.2023.100691 PMC10684810 38033759

[53] Majercakova K, et al. Role of spatially fractionated radiotherapy (LATTICE) treatment in inoperable bulky soft-tissue sarcomas. Cancers. 2025;17(4):624. 10.3390/cancers17040624. 40002219 10.3390/cancers17040624 PMC11853162

[54] Prezado Y, Jouvion G, Patriarca A, et al. Proton minibeam radiation therapy widens the therapeutic index for high-grade gliomas. Sci Rep. 2018;8(1):16479. 10.1038/s41598-018-34796-8. 30405188 10.1038/s41598-018-34796-8 PMC6220274

[55] Iori F, Trojani V, Zamagni A, et al. Spatially fractionated radiation therapy for palliation in patients with large cancers: a retrospective study. Adv Radiat Oncol. 2024;10(1):101665. 10.1016/j.adro.2024.101665. 39687474 10.1016/j.adro.2024.101665 PMC11647081

[56] Wang F, et al. Lattice radiation therapy plays a synergistic role in the radical treatment of bulky cervical cancer: a case report and literature review. Curr Oncol. 2026;33(4):196. 10.3390/curroncol33040196. 42041715 10.3390/curroncol33040196 PMC13114739

[57] Studer G, Streller T, Jeller D, et al. Diagnosis-related outcome following palliative spatially fractionated radiation therapy (lattice) of large tumors. Cancers (Basel). 2025;17(17):2752. 10.3390/cancers17172752. 40940850 10.3390/cancers17172752 PMC12427535

[58] Zhu YN, Zhang W, Lin Y, Gao H. Equivalent-uniform-dose optimization for spatially fractionated radiation therapy. Technol Cancer Res Treat. 2025;24:15330338251380609. 10.1177/15330338251380609. 41134722 10.1177/15330338251380609 PMC12569369

[59] Hosseinian S, et al. Geometric optimization of dose distribution in spatially fractionated radiation therapy. Phys Med Biol. 2025;70(16). 10.1088/1361-6560/adf58f. 10.1088/1361-6560/adf58f 40730217

[60] Zhao H. Progress of the application of spatially fractionated radiation therapy in palliative treatment of tumors. Discov Oncol. 2025;16(1):678. 10.1007/s12672-025-02487-2. 40329010 10.1007/s12672-025-02487-2 PMC12055688

[61] Fang Z, et al. Minibeam radiation therapy remodels tumor vasculature and alleviates radiation-associated hypoxia in triple-negative breast cancer. npj Precis Oncol. 2025. 10.1038/s41698-025-01178-z. 41413184 10.1038/s41698-025-01178-z PMC12714760

[62] Mulyadi R, et al. Dynamic contrast-enhanced magnetic resonance imaging parameter changes as an early biomarker of tumor responses following radiation therapy in lung cancer. Radiat Oncol J. 2023. 10.3857/roj.2023.00094. 38185927 10.3857/roj.2023.00290 PMC10772591

[63] Wang D, Liu S, Fu J, et al. Correlation of Ktrans derived from dynamic contrast-enhanced MRI with treatment response and survival in locally advanced NSCLC patients undergoing induction immunochemotherapy and concurrent chemoradiotherapy. J Immunother Cancer. 2024;12(6):e008574. 10.1136/jitc-2023-008574. 38910009 10.1136/jitc-2023-008574 PMC11328668

[64] Senturk YE, et al. Predictive value of early DCE and DSC perfusion MRI parameters for midterm response in lung carcinoma brain metastases treated with stereotactic radiosurgery. J Neurooncol. 2025. 10.1007/s11060-025-05054-5. 40408063 10.1007/s11060-025-05054-5 PMC12198322

[65] Rivera JN, Laemont K, Tovmasyan A, et al. Minibeam spatially- fractionated radiation therapy is superior to uniform dose radiation therapy for abscopal effect when combined with PD-L1 checkpoint inhibitor immuno therapy in a dual tumor murine mammary carcinoma model. Radiation. 2025;5(1):3. 10.3390/radiation5010003.

[66] Tubin S, Popper HH, Brcic L. Novel stereotactic body radiation therapy (SBRT)-based partial tumor irradiation targeting hypoxic segment of bulky tumors (SBRT-PATzHY): improvement of the radiotherapy outcome by exploiting the bystander and abscopal effects. Radiat Oncol. 2019;14:21. 10.1186/s13014-019-1227-y. 30696472 10.1186/s13014-019-1227-y PMC6352381

[67] Asur RS, Sharma S, Chang CW, et al. Spatially fractionated radiation induces cytotoxicity and changes in gene expression in bystander and radiation adjacent murine carcinoma cells. Radiat Res. 2012;177(6):751–65. 10.1667/RR2780.1. 22559204 10.1667/rr2780.1 PMC3395590

[68] Autsavapromporn N, Kobayashi A, Liu C, et al. Primary and secondary bystander effects of proton microbeam irradiation on human lung cancer cells under hypoxic conditions. Biology (Basel). 2023;12(12):1485. 10.3390/biology12121485. 38132311 10.3390/biology12121485 PMC10741139

[69] Lukas L, Zhang H, Cheng K, Epstein A. Immune priming with spatially fractionated radiation therapy. Curr Oncol Rep. 2023;25(12):1483–96. 10.1007/s11912-023-01473-7. 37979032 10.1007/s11912-023-01473-7 PMC10728252

[70] Schneider T, Fernandez-Palomo C, Bertho A, et al. Combining FLASH and spatially fractionated radiation therapy: the best of both worlds. Radiother Oncol. 2022;175:169–77. 10.1016/j.radonc.2022.08. 35952978 10.1016/j.radonc.2022.08.004

[71] Diffenderfer ES, Verginadis II, Kim MM, et al. Design, implementation, and in vivo validation of a novel proton FLASH radiation therapy system. Int J Radiat Oncol Biol Phys. 2020;106(2):440–8. 10.1016/j.ijrobp.2019.10. 31928642 10.1016/j.ijrobp.2019.10.049 PMC7325740

[72] Ferini G, Parisi S, Lillo S, et al. Impressive results after metabolism- guided lattice irradiation in patients submitted to palliative radiation therapy: preliminary results of LATTICE_01 multicenter study. Cancers (Basel). 2022;14(16):3909. 10.3390/cancers14163909. 36010902 10.3390/cancers14163909 PMC9406022

[73] Fernandez-Palomo C, et al. Should peak dose be used to prescribe spatially fractionated radiation therapy?—a review of preclinical studies. Cancers. 2022;14(15):3625. 10.3390/cancers14153625. 35892895 10.3390/cancers14153625 PMC9330631

[74] Hobson J, Grams MP, Gicobi JK, et al. Case report: complete pathologic response in advanced melanoma with SFRT and dual checkpoint inhibition. Front Oncol. 2025;15:1697902. 10.3389/fonc.2025.1697902. 41179680 10.3389/fonc.2025.1697902 PMC12575138

[75] Deshpande SR, Podder TK, Grubb W, et al. Pretreatment and Posttreatment Tumor Metabolic Activity Assessed by FDG-PET/CT as Predictors of Tumor Recurrence and Survival Outcomes in Early-Stage Non-Small Cell Lung Cancer Treated With Stereotactic Body Radiation Therapy. Adv Radiat Oncol. 2023;9(1):101313. 10.1016/j.adro.2023.101313. 38260218 10.1016/j.adro.2023.101313 PMC10801655

[76] Sathishkumar S, et al. The impact of TNF-alpha induction on therapeutic efficacy following high-dose spatially fractionated (GRID) radiation. Technol Cancer Res Treat. 2002;1(5):349–54. 10.1177/153303460200100509. 10.1177/153303460200100207 12622521

[77] Deng L, et al. STING-dependent cytosolic DNA sensing promotes radiation- induced type I interferon-dependent antitumor immunity. Immunity. 2014;41(5):843–52. 10.1016/j.immuni.2014.10.004. 25517616 10.1016/j.immuni.2014.10.019 PMC5155593

[78] Mathieu M, et al. Activation of STING in response to partial-tumor irradiation. Int J Radiat Oncol Biol Phys. 2023. 10.1016/j.ijrobp.2023.05.021. 37244631 10.1016/j.ijrobp.2023.05.032 PMC11334988

[79] Zhu L, et al. Simulation-free workflow for lattice radiation therapy using deep learning predicted synthetic computed tomography: a feasibility study. J Appl Clin Med Phys. 2025. 10.1002/acm2.14600. 40504103 10.1002/acm2.70137 PMC12256696

[80] Gaudreault M, Chang D, Kron T, et al. Development of an automated treatment planning approach for lattice radiation therapy. Med Phys. 2024;51:682–93. 10.1002/mp.16761. 37797078 10.1002/mp.16761

[81] Mayr NA, et al. An international consensus on the design of prospective clinical–translational trials in spatially fractionated radiation therapy. Adv Radiat Oncol. 2022;7(2):100866. 10.1016/j.adro.2021.100866. 35198833 10.1016/j.adro.2021.100866 PMC8843999

