# Supplementary material for: Spatially fractionated radiation therapy (SFRT): a scoping review of dosimetric and biological evidence supporting a valley dose–driven translational framework
Source: Radiat Oncol. 2026 Jun 4;21:104. doi: 10.1186/s13014-026-02865-2 (PMC13455314; doi:10.1186/s13014-026-02865-2)
Supplement: Supplementary file 1 [file 13014_2026_2865_MOESM1_ESM.doc]

**Supplementary Tables**

**Appendix Table S1: Complete Search Strategies for Each Database**

(Search Timeframe: January 1, 2005–July 1, 2025; Search fields cover Title and Abstract for all databases; Subject terms/expanded search are used for specific databases to improve accuracy)

| **Database Name** | **Search Field/Grammar Description** | **Complete Search String** |
| --- | --- | --- |
| PubMed | Combines MeSH Terms and Title/Abstract fields ([MeSH Terms] indicates subject term search; [Title/Abstract] indicates field limitation) | ("Spatially Fractionated Radiation Therapy"[Title/Abstract] OR "SFRT"[Title/ Abstract] OR "Grid Therapy"[Title/Abstract] OR "Lattice Radiotherapy" [Title/Abstract] OR "Minibeam Radiotherapy" [Title/Abstract] OR "Microbeam Radiotherapy" [Title/Abstract] OR "Proton SFRT"[Title/Abstract]) AND ("Neoplasms"[MeSH Terms] OR "tumor"[Title/Abstract] OR "cancer" [Title/Abstract]) AND ("Dosimetry" [Title/Abstract] OR "Radiobiology" [Title/Abstract] OR "Clinical Trial" [pt]) AND ("2005/01/01"[PDAT] : "2025/07/01"[PDAT]) |
| Embase | Uses Embase expanded subject search (/exp/ indicates expanded search) and Title/Abstract fields (/ti,ab/) | /exp/spatially fractionated radiotherapy/ OR "sfrt"/ti,ab/ OR "grid therapy"/ti,ab/ OR "lattice radiotherapy"/ti,ab/ OR "minibeam radiotherapy"/ti,ab/ OR "microbeam radiotherapy"/ti,ab/ OR "proton sfrt"/ti,ab/ AND /exp/neoplasm/ OR "tumor"/ti,ab/ OR "cancer"/ti,ab/ AND "dosimetry"/ti,ab/ OR "radiobiology"/ti,ab/ OR "clinical trial"/ti,ab/ AND 2005-2025/yr |
| Web of Science | Takes "Topic" (TS=) as the core search field, covering Title, Abstract, and Keywords | TS=("Spatially Fractionated Radiation Therapy" OR "SFRT" OR "Grid Therapy" OR "Lattice Radiotherapy" OR "Minibeam Radiotherapy" OR "Microbeam Radiotherapy" OR "Proton SFRT") AND TS=("Neoplasms" OR "tumor" OR "cancer") AND TS=("Dosimetry" OR "Radiobiology" OR "Clinical Trial") AND PY=2005-2025 |
| Cochrane Library | Adapts to the search format of Cochrane Central Register of Controlled Trials, focusing on clinical study relevance | ("Spatially Fractionated Radiation Therapy" OR "SFRT" OR "Grid Therapy" OR "Lattice Radiotherapy" OR "Minibeam Radiotherapy" OR "Microbeam Radiotherapy" OR "Proton SFRT") AND ("Neoplasm" OR "Tumor" OR "Cancer") AND ("Dosimetry" OR "Radiobiology" OR "Randomized Controlled Trial" OR "Clinical Study") AND (2005 TO 2025) |

Appendix Table S2: Modified JBI Appraisal Framework for SFRT Evidence Mapping (Scoring: 1 = Met, 0 = Not Met; High Quality: ≥ 70%, Moderate: 50%–69%, Low: < 50%)

| **Category** | **Appraisal Dimension** | **Specific Scoring Criteria (SFRT-Specific)** |
| --- | --- | --- |
| Preclinical  (7 Points) | 1. MBO Logic | Does the study explicitly correlate spatial dose gradients with specific immune/molecular signatures? |
|  | 2.Dosimetric Rigor | Is the delivered Dvalley and Dpeakverified via independent detectors (e.g., GAF film)? |
|  | 3. Allocation | Is animal or cell-line grouping randomized with a clearly described methodology? |
|  | 4. Blinding | Were biological outcomes (e.g., IHC scoring) assessed by observers blinded to the dose groups? |
|  | 5.Experimental Power | Are at least 3 independent replicates reported to ensure biological reproducibility? |
|  | 6. Ethical Compliance | Is IACUC approval or cell line authentication (STR) explicitly documented? |
|  | 7. Statistical Fit | Are the statistical methods appropriate for the variance observed in microenvironmental data? |
| Clinical  (7 Points) | 1. Technical Fidelity | Are SFRT modality, beam energy, and the definition of Dvalley prescription clearly reported? |
|  | 2. Surrogate Validity | Is the link between early response markers (e.g., PET/MRI) and clinical outcomes analyzed? |
|  | 3. Baseline Control | Are prior radiotherapy history (re-irradiation) and tumor histologies explicitly accounted for? |
|  | 4. Toxicity Grading | Is acute and late toxicity systematically graded according to CTCAE v5.0 or equivalent? |
|  | 5. Longitudinal Data | Is the follow-up duration sufficient to capture the "5-year local control" proposed in the model? |
|  | 6. Ethical Integrity | Is IRB approval and patient informed consent documented? |
|  | 7. Response Criteria | Is the clinical efficacy evaluated using standardized criteria (e.g., RECIST 1.1)? |
| Technical  (6 Points) | 1. Grid Resolution | Is the dose calculation grid size (≤ 1mm) sufficient to resolve steep Dvalley gradients? |
|  | 2. 3D Geometry | Does the study provide tumor volume or depth data to support the 3D Technique Selection Model? |
|  | 3. Algorithm Fidelity | Is a high-fidelity algorithm (e.g., Monte Carlo, Collapsed Cone) utilized for Dvalley modeling? |
|  | 4.Cross-Verification | Is the dosimetric accuracy cross-verified (e.g., Calculation vs. Experimental measurement)? |
|  | 5.Uncertainty | Are dosimetric metrics reported with associated error margins or gamma pass rates? |
|  | 6. Protocol Detail | Is the collimator/aperture design or delivery workflow detailed enough for replication? |

**Appendix Table S3:. Distribution of the 60 Landmark Studies by Study Type** (16 Preclinical + 26 Clinical + 18 Technical/Dosimetric)

| **Study Type related** | **Number** | **Representative Literature Examples (Ref Numbers, All Listed)** | **Main Contribution to the Dvalley-centric Framework** |
| --- | --- | --- | --- |
| Preclinical Studies (Animal/In Vitro Mechanisms) | 16 | [4,11,12, 20, 33, 34, 45, 54, 61, 65-68, 70,71,73] | Provides evidence of Dvalley-mediated vascular/immune/bystander/FLASH biological cascades |
| Clinical Studies (Human Cohorts /Trials/Outcomes) | 26 | [1, 3, 10, 13, 14,18, 25, 27，32, 35, 36, 39, 40, 42, 47, 48, 49, 52, 53, 55- 57, 72, 74-76] | Directly validates the safety, ORR, and local control rates of valley dose in bulky tumors |
| Technical/Dosimetric Reports (Planning/Optimization/Dosimetry) | 18 | [6, 7, 9, 21- 24, 26, 28-30, 38, 44, 46, 51, 58-60] | Supports Dvalley precision optimization, PVDR control, and standardized roadmap for future trials |
